# The structure differences of Japanese encephalitis virus SA14 and SA14-14-2 E proteins elucidate the virulence attenuation mechanism

**DOI:** 10.1007/s13238-018-0551-6

**Published:** 2018-05-11

**Authors:** Xinyu Liu, Xin Zhao, Rui Na, Lili Li, Eberhard Warkentin, Jennifer Witt, Xu Lu, Yongxin Yu, Yuquan Wei, Guohong Peng, Yuhua Li, Junzhi Wang

**Affiliations:** 1 National Institutes for Food and Drug Control, Beijing, 102609 China; 2 Department of Molecular Membrane Biology, Max Planck Institute of Biophysics, 60438 Frankfurt am Main, Germany; 3 State Key Laboratory of Biotherapy and Cancer Center, West China Hospital, Sichuan University, and Collaborative Innovation Center for Biotherapy, Chengdu, 610041 China

**Dear Editor,**

Japanese encephalitis (JE) is a mosquito-borne acute neurological infectious disease caused by the Japanese encephalitis virus (JEV). Globally, 68,000 cases of the disease are estimated each year, with a fatality rate of as high as 30% and with approximately 30%–50% of survivors suffering from severe neurological sequelae (WHO, 2015).

Vaccination is the most economical and effective method for preventing JE infections. JE live vaccine SA14-14-2 is the most widely used vaccine being utilized in many countries such as China, South Korea, Nepal, India and Thailand etc., since its introduction to the market in 1989. JE attenuated virus SA14-14-2 was derived from its parental virulent virus SA14, and its safety was demonstrated in animals and over 800 million doses use in humans (Yu, 2010). However, the underlying mechanism of JE live vaccine SA14-14-2 attenuation mechanism remains unclear.

Studies have demonstrated that E protein is the most important protein determing the virulence of JEV (Yang et al., 2017). A comparison of the full genome sequences of JEV SA14 and SA14-14-2 revealed 57 different nucleotides and 24 amino acid changes. Eight of them were found in the E protein (Table S1) (Aihara et al., 1991). Some amino acid mutations were discovered to be crucial for virus virulence (Yang et al., 2014), but how they affect the viral virulence remained unclear. To address this open question we determined the X-ray structures of the E protein ectodomains of JEV SA14-14-2 and of its parent virulent SA14 virus in a monomeric state and reliably modeled dimers (assembled to 180-mers) and trimers mostly present during the viral life cycle. The aim was to elucidate theoretical mechanism for the practically established safety of JE live vaccine SA14-14-2 and help with the quality control and development of flavivirus vaccines.

The overall crystal structures of JEV SA14 and SA14-14-2 E proteins were determined at a resolution of 2.2 Å and 2.1 Å, respectively, by molecular replacement methods (Table S2). The E protein sequence of JEV SA14-14-2 in our study differs from that of JEV SA-14-14-2 served as model (PDB ID: 3P54) (Luca et al., 2012) by two different amino acids at positions 177 and 264. The JEV SA14-14-2 in our study is used for the JE live attenuated vaccine production in China. However, there is no information on the passaging history and cell substrate of the virus cultivation of JE virus SA-14-14-2 recorded in PDB ID: 3P54. The structures of JEV SA14 and SA14-14-2 E protein are highly similar to those of other flaviviruses. Architecturally, all of them consist of three domains, Domain I (DI, yellow), Domain II (DII, red) and Domain III (DIII, dark blue) (Fig. 1A). JEV SA14 and SA14-14-2 E protein ectodomains are distinguished by seven mutations (listed in the Table S1).

**Figure 1:**
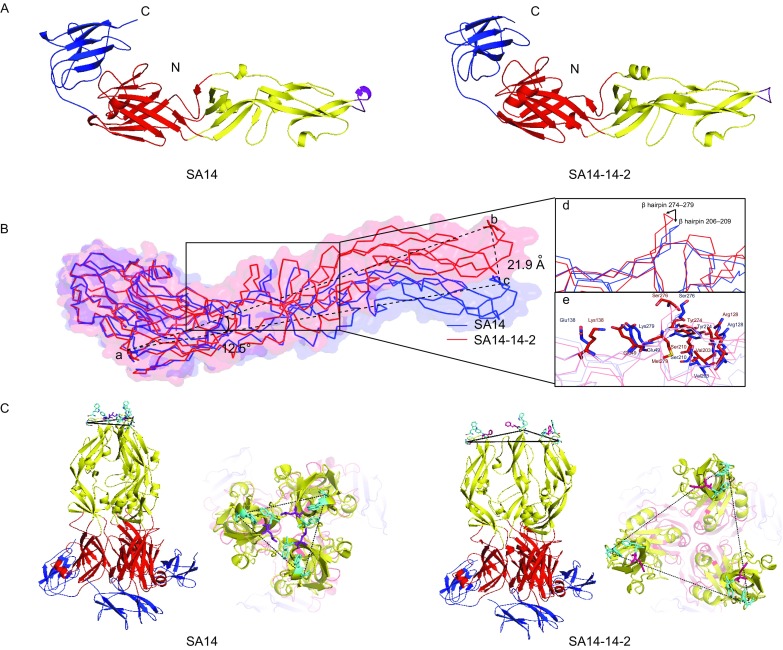
**Structural comparisons of E protein ectodomain between JEV SA14 and SA14-14-2**. (A) Cartoon views of crystal structures of JEV SA14 and SA14-14-2. (B) Conformational comparison between JEV SA14 and SA14-14-2. DIII is considered as rigid body for superimposing structures of JEV SA14 (blue) and SA14-14-2 (red). Asn36-C_α_ is point a, Gly102-C_α_ of JEV SA14-14-2 is point b, Gly102-C_α_ of SA14 is point c, ∠bac is 12.5° and distance between b and c is 21.9 Å. Magnified views of a and b are formed by rotating highlight square view (20°), for clearly displaying the variations of the β hairpin and residue orientations. d: A magnified view of the conformational shifts at the β hairpin 274–279 and 206–209 of JEV SA14 and SA14-14-2 E protein. e: A more detailed view of residues Glu138Lys and Lys279Met surroundings in JEV SA14 and SA14-14-2 E protein. The major difference represents the distinct orientation of DII relative to DI + DIII due to the Glu138Lys and Lys279Met mutations located in the hinge region. (C) The trimeric structure comparisons of JEV SA14 and SA14-14-2 E proteins. From left to right are lateral views of JEV SA14, top views of fusion loops of JEV SA14, lateral views of SA14-14-2 trimers, top views of fusion loops of JEV SA14-14-2. The trimer model is derived from the TBV E protein

Structural comparison between JEV SA14 and SA14-14-2 E protein ectodomains revealed a rotation of DI and DIII relative to DII (Fig. 1B). The rotation angle between JEV SA14 and SA14-14-2 DII relative to the superimposed DIII was 12.5° resulting in a distance between the two Gly102 (b and c) of 21.9 Å. The hinge points for the relative rotation are located close to the distinct amino acids at positions 138 and 279 (Fig. 1B green dot and orange dot).

Interestingly, the side chain of JEV SA14 Lys279 protrudes to the bulk solvent whereas that of JEV SA14-14-2 Met279 is directed to the protein interior (Fig. S1A and S1B). Notably, JEV SA14-14-2 Met279 superimposes with JEV SA14 Val280 which implicates a shortcut of segment 280–283 and a prolongation of the preceding β hairpin 274–279 (Fig. 1B d and e). Met279 contacts Ala50, Ile130, Val203, Val272 and Tyr274 and thus becomes part of a hydrophobic patch which is larger than that in JEV SA14 E protein ectodomain (Fig. S1C and S1D). Due to the rearrangement of segment 274–283 Val278 and Leu280 are turned towards the solvent and form together with the nonpolar part of Glu49 a small hydrophobic patch (Fig. S2A and S2B). The prolongation of the β hairpin 274–279 causes a further extension into the bulk solvent and a rotation by approximately 10° towards the DI side of the hinge (Fig. 1B d and e). DI and DII are only linked by the β hairpin 274–279, its conformation determines the orientation of the entire DII.

While Glu138Lys change is the key event to shift Glu49 to Lys138 in JEV SA14-14-2 E protein and thus induce the inversion of the side chain direction of residue 279 from the bulk solvent to the hydrophobic protein interior (Fig. S1D) accompanied by a rotation of the preceding β hairpin 274–279 towards DIII which pulls the entire DII with it (Fig. 1B d and e). The Lys279Met change does, apparently, not induce the reorientation of the side chain which only occurs cooperatively with the Glu138Lys mutation. Creating revertant viruses and subsequent neurovirulence test on mice demonstrated a major effect of residue 138 and cooperatively effect of residue 279 (Yang et al., 2017). In our study, the revertant viruses with Phe107Leu and Lys138Glu resulted in 50% and 30% mortality in mice, respectively, whereas revertant virus with two amino acids mutations of Lys138Glu/Met279Lys and SA14 exhibited 100% mortality in mice. And revertant viruses with amino acids mutations of Met279Lys, Val315Ala and JE attenuated virus SA14-14-2 inoculation caused 0% mortality in mice (Fig. 2A).

**Figure 2:**
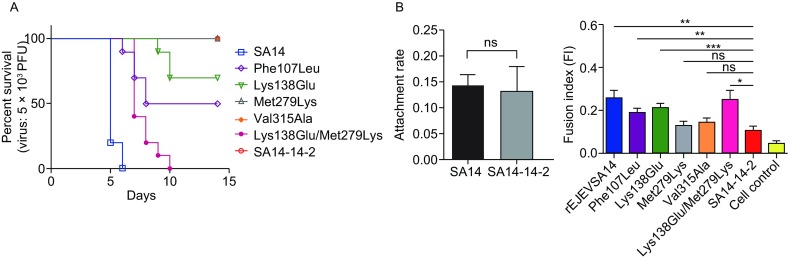
**Neurovirulence, binding affinity and fusion activity of JEV SA14, SA14-14-2 and the revertant viruses**. (A) Binding affinity of JEV SA14 and SA14-14-2 to BHK_21_ cells. (B) Fusion activity of JEV SA14-14-2 and revertant viruses measured by the fusion index in BHK_21_ cells. Quintuplicate wells were used for cells infection by each of JEV SA14-14-2 and revertant viruses and the uninfected BHK_21_ cells were used as control. Data are represented as mean ± SEM. *P < 0.05; **P < 0.01; ***P < 0.005, ns, not significant. (C) Neurovirulence of JEV SA14, SA14-14-2 and revertant viruses in mice

Besides the Glu138Lys, the Leu107Phe change is the most important which is substantiated by revertant virulence tests of JEV SA14-14-2 (Fig. 2A). Residue 107 is part of the fusion loop which consists of 10 amino acids (positions 100–109) and is located on the tip of DII of the E protein. Although the fusion loops of the determined JEV SA14 and SA14-14-2 E protein are structurally similar, the Leu107Phe change slightly modifies the surface profile and the hydrophobicity (Fig. S3). The difference might be important because the side chains of Trp101, Leu107 and Phe108 conserved among all flaviviruses form the hydrophobic anchor that can invade into cell membrane of the target (Harrison, 2015). Other amino acid changes (Table S1) does not appear to significantly change the structure of JEV SA14-14-2 compared to JEV SA14. Changes of residues 176/177/264/315 have no influence on the virulence attenuation which agrees with virulence experiment.

Due to the lack of experimental data we calculated the interface between two monomers for JEV SA14 and SA14-14-2 E protein ectodomain using PISA. Accordingly, JEV SA14-14-2 monomer provides a plausible dimer (complexation significance score (CSS), 1.00) with a nearly two-fold axis while JEV SA14 monomer cannot pack to a reliable dimer (CSS, 0.00) (Table S3). For modelling an E protein dimer we used the experimentally determined TBV E protein dimer as an esamble. The area and hydrophobicity of the DII fusion loop-DIII interface of JEV SA14 dimer is decreased with the fusion loop slightly shifting away from the hydrophobic cavity in comparison with that of JEV SA14-14-2 dimer (Fig. S4). All together, the smaller overall contact area and number of interactions and the requirement of significant rearrangements argue for a weaker monomer-monomer affinity of JEV SA14 compared to JEV SA14-14-2 E protein dimer.

JEV SA14 and SA14-14-2 E protein trimers were generated by superimposing three DI onto the TBV E trimer (PDB ID: 1URZ) (Bressanelli et al., 2004) (Fig. 1C). The rmsd between DI and DIII of JEV SA14 and TBV E protein are 2.7 Å and 1.8 Å (Table S3). Due to the different DI to DII orientation of the monomer, the distance between the same residue 104 of the fusion loop increases from 25.2 Å in JEV SA14 to 46.5 Å in JEV SA14-14-2 trimers, respectively (Fig. 1C). The rotation angle between the DII domains in JEV SA14-14-2 trimer is ca. 24° (Fig. 1C). The three elongated DII domains are tightly packed in JEV SA14 trimers and separately arranged in JEV SA14-14-2 trimers. The latter appears therefore to be more mobile. Only in the compact JEV SA14 trimer assembly, the three conserved residues Trp101, Leu107 and Phe108 of the fusion loop form a bowl-like hydrophobic cavity. Leu107 is strictly conserved in flaviviruses and the importance of the fusion loop for their virulence is experimentally demonstrated (Huang et al., 2010; Zhang et al., 2006).

Flavivirus infects a host cell by endocytosis by which DIII of the exposed E protein of the entire virus particle binds to the cell-surface receptor of the host. The 180-meric E protein becomes disassembed via dimers into monomers upon acid pH of the endosomal vesicle (Mukhopadhyay et al., 2005). According to the established structural data, we assumed the dimer formation/dissociation energy is higher and thus the disintegration slower for JE attenuated virus SA14-14-2 than for JE virulent virus SA14 E protein which might attenuate viral virulence. Subsequently, the monomers irreversibly pack to trimers on the virion surface (Modis et al., 2004) with the fusion loops being exposed at the tip. The found separated arrangement of the three fusion loops in JE attenuated virus SA14-14-2 E protein trimer acting in a concerted manner in the compact JE virulent virus SA14 trimer might decrease the membrane fusion efficiency. According to the current knowledge the indole amine side chain of Trp101 enters the hydrocarbon layer of the host cell membrane but the hydrophobic bowl cannot accommodate lipid head groups (Modis et al., 2004). We speculated the aromatic side chain of Phe107 in JE attenuated virus SA14-14-2 E protein might cause some steric hindrance, such that the invasion of Trp101 of JE attenuated virus SA14-14-2 E protein into the membrane is hampered compared to JE virulent virus SA14 thereby causing virulence attenuation.

Next, the virus completely disassembles and the viral genome is released into the cytoplasm of the host cell. After replication of genome and expression of structural and nonstructural proteins, viruses are assembled on the surface of the endoplasmic reticulum (ER). The resulting immature virion is then transported into the trans-Golgi network, subjected to a maturation process and finally released from the host cell by exocytosis. Interestingly, different maturation states of the E proteins of DEN sE (P) during the viral life cycle are characterized by a different DI + DIII relative to DII angle (Zhang et al., 2004). From our research, the DII relative to DI + DIII orientation appears to be controlled by the state of the hydrophobic pocket (in front of Met279 in JE attenuated virus SA14-14-2 E protein, Fig. S1D). It can be open and closed by binding a ligand or by adjusting the pH threshold for fusion and thus reorient the crucial β hairpin as reported for the Dengue sE (Modis et al., 2003).

It might be speculated that the transition between different DI + DIII to DII orientations necessary for maturation is impeded and thus virulence is attenuated in JE attenuated virus SA14-14-2 E protein due to the inversion of the residue at position 279 mainly triggered by the Glu138Lys change. In this context it is of relevance that the structure of another JEV SA-14-14-2 E protein (PDB ID: 3P54, space group I222) (Luca et al., 2012) reveals a nearly identical DI + DIII relative to DII orientation (overall r.m.s.d. 0.9 Å) indicating that the angle is independent of crystal packing and the found conformation apparently very rigid.

The binding affinity of JEV SA14 and SA14-14-2 to the host cells is similar (Fig. 2B), so the binding affinity is not the main cause of JEV SA14-14-2 neurovirulence attenuation.

A cell-cell fusion assay was performed to compare the fusion activity of JEV SA14-14-2 and its revertant viruses with different amino acids mutations in E protein from SA14-14-2 to SA14. The results (Fig. 2C) showed that JEV SA14-14-2 has the lowest fusion activity and recombinant virus rJEVSA14 with E protein of SA14 based on the backbone of JE attenuated virus SA14-14-2 genome had the highest fusion activity. Revertant virus Phe107Leu and Lys138Glu had the significantly higher fusion activity than that of SA14-14-2. There was no significant difference between the viruse Val315Ala and SA14-14-2, which coincided with our neurovirulence results (Fig. 2A) and Dr. Wang’s studies (Wang et al., 2017). Although the fusion activity of revertant viruses with Met279Lys was not significantly higher than that of SA14-14-2, revertant virus with two amino acids Lys138Glu/Met279Lys combined was furtherly higher than that of SA14-14-2. It demonstrated that residues 138 and 279 cooperatively changed the fusion activity of JEV SA14-14-2 which also coincided with our neurovirulence results (Fig. 2A).

The lower fusion activity of JE attenuated virus SA14-14-2 is compatible with its unfavorable trimer geometry and its reduced dimer dissociation capability because of the fixation of the DII to DI + DIII angle in JEV SA14-14-2 monomers due to the mutation of residues 107, 138 and 279. And the reduced fusion indexes might be responsible for the attenuation of JE attenuated virus SA14-14-2.

The presented data implicate that the virulence attenuation of JE attenuated virus SA14-14-2 has its structural foundations due to the cooperative effects of amino acids at several positions of the E protein. It is considered as unlikely that the JE live vaccine SA14-14-2 reverts into its parental virus virulence by random amino acids mutations. Thus JE live vaccine SA14-14-2 is genetically stable and safe. Moreover, the structural data might serve as a guide for the quality control and research and development of other new flavivirus vaccines. But because the dimer and trimer structures were modelled, native oligomeric structure were needed to furtherly verify the conclusion.

## Footnotes

The authors would thank Hartmut Michel for great supports on performing crystallography works at the Max Planck Institute of Biophysics. We are grateful to Ulrich Ermler (Max Planck Institute of Biophysics) for scientific discussion on crystallography and critical reading the manuscript, thank Barbara Rathmann, David Quirnheim Pais and Yvonne Thielmann for technique assistance during automated protein crystallization. We thank Xiangxi Wang (Institute of Biophysics, CAS), and Guangwen Lu (West China Hospital, Sichuan University) for their scientific suggestion with the research. We thank the NCPSS, SSRF and SLS for provision of synchrotron facilities. We also thank the staffs of beamlines BL18U1/BL19U1 (NCPSS), BL17U1 (SSRF) and X10SA (SLS) for assistance during data collection. This work was supported by China, Grant No. 2014ZX09304316-003 and Grant No. 2015DFA30690), and Max-Planck-Gesellschaft.

Xinyu Liu, Xin Zhao, Rui Na, Lili Li, Eberhard Warkentin, Jennifer Witt, Xu Lu, Yongxin Yu, Yuquan Wei, Guohong Peng, Yuhua Li and Junzhi Wang declare that they have no conflict of interest. All institutional and national guidelines for the care and use of laboratory animals were followed.

## Electronic supplementary material

Below is the link to the electronic supplementary material.

Supplementary material 1 (PDF 640 kb)
