# Supplementary material for: The structure differences of Japanese encephalitis virus SA14 and SA14-14-2 E proteins elucidate the virulence attenuation mechanism
Source: Protein Cell. 2018 May 11;10(2):149–53. doi: 10.1007/s13238-018-0551-6 (PMC6340892; doi:10.1007/s13238-018-0551-6)
Supplement: Supplementary file 1 — Supplementary material 1 (PDF 640 kb) [file 13238_2018_551_MOESM1_ESM.pdf]

Supplementary Material

Methods

Cloning, expression, and purification of JEV E proteins

The viral RNA of SA14 and SA14-14-2 (stored in NIFDC) was extracted(Qiagen) and used as templates for reverse transcription synthesis of cDNA (Promega). PCR was used to amplify the gene sequences of the 406 amino acids of the E protein ectodomain. The target gene was constructed in a modified prokaryotic expression vector, pQEC3 (Provided by the Max Planck Institute of Biophysics) and transfected into competent BL21 cells (Tiangen). The sequence was verified through gene sequencing (Invitrogen). IPTG (Ameresco) was used to induce expression of the target protein and sodium dodecyl sulfate-polyacrylamide gel electrophoresis was used to verify that the expressed products were mainly comprised of inclusion bodies. The rapid dilution method was used to perform inclusion body refolding as previously described[1,2]. Briefly, the inclusion bodies were dissolved using 80 ml of the inclusion body solvent (6M guanidine-HCl, 10 mM Tris [pH 7.8], 20 mM β-mercaptoethanol). The solution was then added dropwise at a specific rate into 8 l of rapidly stirred refolding buffer (400 mM nondetergent sulfobetaine-201, 100 mM Tris [pH 7.8], 0.5 mM oxidized glutathione, 5 mM reduced glutathione, 5% glycerol), and the refolding process was performed overnight at 4°C. The refolded protein solution was concentrated via ultrafiltration using 10 kD pore size membranes (Millipore, Sartorius) and then purified using gel-filtration chromatography (GE Healthcare), to obtain purified E proteins. Mass spectrometry (MicrOTOF-QII (BrukerDaltonics)) was used to determine whether the expressed E protein was the target protein.

Crystallization of JEV E proteins

Standard crystallization screening was carried out using an ARI Gryphon dispenser or a CrystalMation robot system (Rigaku, USA). JEV E proteins with a concentration of 6 mg/ml were dropped onto a 96-well plate. The drop consisted of 0.2 μl protein solution and 0.2 μl mother liquor, followed by the addition of 60 μl mother liquor to each well. Crystallization screens were purchased from Hampton Research (USA), Sigma (USA), Jena Bioscience (Germany) and Qiagen (Germany). Crystallization was then performed at 18°C. After the appropriate mother liquor conditions for crystal growth were selected, vapor diffusion in sitting drop plate (24-wells) was used to reproduce the crystals. Crystals of SA14 E protein appeared after 5 days, mixing a 5μl aliquot of protein (6 mg/ml) at 1:1 (v/v) ratio with a reservoir solution comprised of 0.1 M Tris-HCI (pH 8.0), 0.3 M Ammonium acetate, 16% (w/v) Polyethylen glycol (PEG) 1000. For crystallization of the live attenuate SA14-14-2 E protein, a 5μl of aliquot of protein (6 mg/ml) at 1:1 ratio was mixed with a reservoir solution composed of 18% (v/v) PEG 3350 and 0.2 M Sodium citrate tribasic dihydrate (pH 7.9), crystal grew within 2 weeks.

Data collection, processing, and structure solution

The crystals were collected and flash-cooled in liquid nitrogen. The cryoprotectant solution used was the crystallization mother liquor containing 25% glycerol.

Both native data sets of SA14 and SA14-14-2 E proteins were collected at beamline BL18U1 of the National Center for Protein Sciences Shanghai (NCPSS) at SSRF, indexed and processed with the program XDS[3] to a resolution of 2.2 and 2.1 Å, respectively. Data collection statistics are listed in Table 2.

Structure solution

The structures were determined employing the Molecular Replacement Method using the native model (PDB ID: 3P54)[1] as an ensemble in the program PHASER[4].

Refinement

The structures were refined initially using REFMAC5[5] and PHENIX REFINE[6] for the final stages. Necessary model improvements as well as search for solvent molecules were performed using COOT[7] and 'update water' in PHENIX REFINE. Anisotropic thermal displacement factors were refined at 2.2 Å or better resolution, otherwise using the TLS model.

Neurovirulence test

Because in the reference[8-12], virulence of some revertant viruses hadn't been assessed, we conducted the neurovirulence test. Groups (n=10) of 12-14g SPF Kunming mice were inoculated with JEV SA14, SA14-14-2 (stored in NIFDC), diluent and revertant viruses (provided by Chengdu Institutes of Biological Products) with one or two amino acid mutations of E protein from JEV SA14-14-2 to SA14 by containing same virus content of 5×10^3 PFU by i.c. route. The mice were observed for 14 days and the numbers of dead and alive mice were recorded. All animal experiments were conducted in strict accordance with the guidelines of the Experimental Animal Welfare and Ethics Committee of the National Institutes for Food and Drug Control.

Virus binding assay

The cell binding affinity of JEV to Baby Hamster Kidney 21(BHK_{21})cells was assessed as previously described[13]. Briefly, BHK_{21} cell sheets were incubated at 4°C for 30 mins with

virus content of 10^5 PFU of JEV SA14 and SA14-14-2 respectively. Viral RNA copies from cell lysates and supernatants were determined by real-time polymerase chain reaction (PCR) amplification. Results are shown as the mean value ± standard difference (SD) from three independent experiments.

Cell-cell fusion assay

The fusion activity of JEV was determined by counting the number of lysed cells after the cells were infected[14-16]. In brief, BHK_{21} cells were first cultured into monolayers in a 24-well plate. Each well was inoculated with 5×10^2 PFU of JEV SA14-14-2 and its revertant viruses with different amino acids mutation in E protein. Then, 20 mmol HEPES was added into the culture medium and the pH was adjusted to 7.8 to allow the virus proliferation in a high pH environment. After incubation at 37°C and 5% CO_2 incubator for 2 days, the medium was replaced by fusion medium buffered with MES to pH 6.3 for 2 hours. Following fusion, the cells were subjected to Giemsa staining. The numbers of cells with and without membrane fusions in each well were counted under a microscope, and the fusion index (FI) was calculated as FI = 1- (number of cells/number of nuclei); at least 5 wells were counted for each virus sample. Results were derived from two independent assays.

Table S1. Amino acid sequence differences of JEV SA14 and SA14-14-2 E proteins

Site of Amino Acid	SA14*	SA14-14-2**
**107**	Leu	Phe
**138**	Glu	Lys
**176**	Ile	Val
**177**	Thr	Ala
**264**	Gln	His
**279**	Lys	Met
**315**	Ala	Val
**439**	Lys	Arg

*SA14 used in the table is the parent virulent strain of JE live attenuated vaccine SA14-14-2.

**SA14-14-2 used in the table was the primary seed for vaccine production. Both the viruses are stored in National Institiutes for Food and Drug Control(NIFDC) where JE live vaccine SA14-14-2 was invented. The sequence had been recorded in the Genbank D90194 and D90195 respectively.[17]

Table S2. Data collection and refinement statistics

**Dataset**	**SA14**	**SA14-14-2**
Data collection		
space group	$P2_1$	C2
cell dimensions a,b,c (Å),	67.09, 33.19, 98.51,	130.60, 55.90, 90.10,
α, β , γ (°)	90.00, 107.46, 90.00	90.00, 132.10, 90.00
temperature (K)	100	100
wavelength (Å)	0.97776	0.97621
resolution range (Å) (high)	20-2.2 (2.3-2.2)	20-2.1 (2.2-2.1)
completeness (%)	98.9 (98.9)	98.7 (98.7)
Redundancy	4.4 (4.6)	6.7 (7.0)
$<I/\sigma I>$	10.2 (0.8)	9.7 (2.8)
Rmeas (%) (high resolution)	15.8 (193.6)	15.6 (94.4)
CC(½) (high resolution)	99.3 (38.1)	99.5 (87.7)
Refinement statistics		
resolution range (high) (Å)	20–2.25 (2.37–2.25)	20 – 2.1 (2.175 – 2.10)
Rcryst (%)	21.1 (33.3)	18.0 (27.5)
Rfree (%)	27.2 (36.1)	22.3 (33.1)
R.m.s. deviation bonds (Å)	0.005	0.012
R.m.s. deviation angles (°)	0.78	1.19
No. of monomers/AU	1	1
No. waters/AU	115	236

Table S3. Comparison of R. m. s. deviations of superimposed structures of JEV SA14 or SA14-14-2 and the other E proteins.

**Specie name**	**SA14 R. m. s. deviations (rmsd)** （**Å**）			
	Monomer of E protein	Domain I	Domain II	Domain III
SA14-14-2	2.5	1.1	1.4	0.5
DENV-Immature(1tg8)	3.7	2.9	1.9	1.2
DENV-Mature(1oke)	3.2	3.0	1.9	1.2
DENV-Post(1ok8)	3.6	2.4	1.5	2.2
TBV(1urz)	3.6	2.7	1.5	1.8
	**SA14-14-2 R. m. s. deviations** （**Å**）			
SA-14-14-2 (3p54)	0.9	0.9	0.7	0.8

Table S4. Analysis of dimer interfaces of JEV SA14 and SA14-14-2 E protein

Envelope	Solvent-accessible area (Å^2)		Solvent-accessible area/Total (%)		Number of Residues		No. of bonds		CSS
	A	B	A	B	A	B	H bonds	Salt bridges	
SA14	473.8	540.6	2.5	2.9	26	16	1	0	0
SA14-14-2	991.2	989.1	5.0	5.0	41	41	4	4	1

Note: A and B represent two monomer of the dimer, respectively. CSS: Complexation significant score

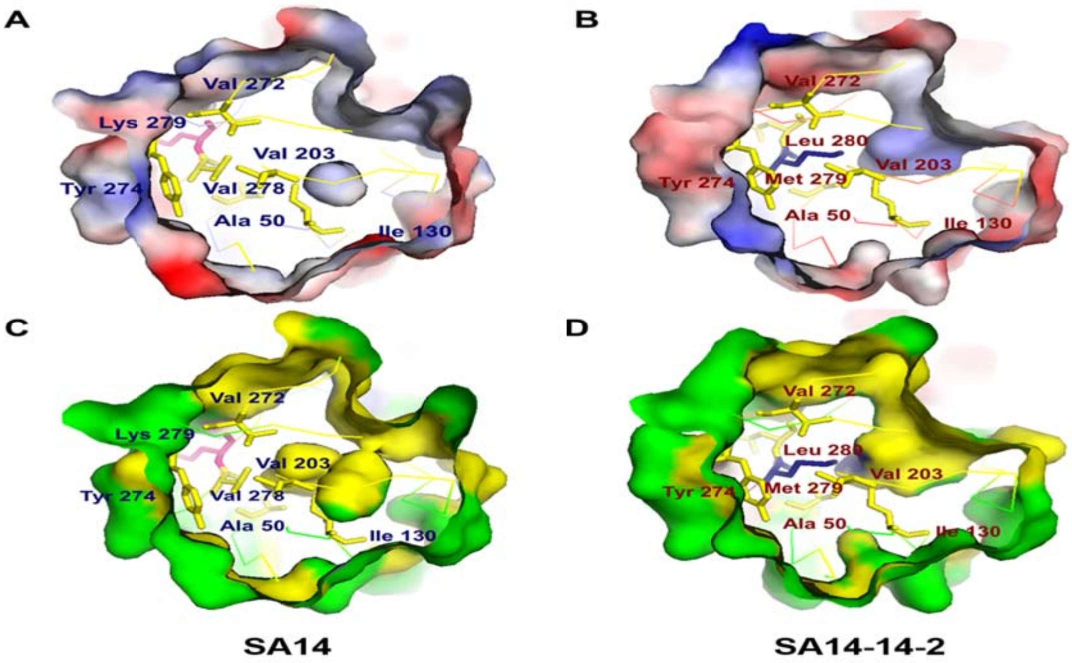

Figure S1. The residue 279 surroundings of JEV SA14 and SA14-14-2 E proteins

(A) A cross-section view of residue Lys279 surroundings of SA14 E protein.

(B) A cross-section view of residue Met279 surroundings of SA14-14-2 E protein. The negative electrostatic potential surface shows in red and positive one in blue. The amino acid at position Lys279 is represented by a pink stick (A), Met279 by dark blue (B) and surroundings residues in yellow.

(C) and (D), The same cross-section views as A and B, show the residue 279 surroundings of SA14 and SA14-14-2, respectively. Hydrophobic surface is shown in yellow. Hydrophilic surface is in green.

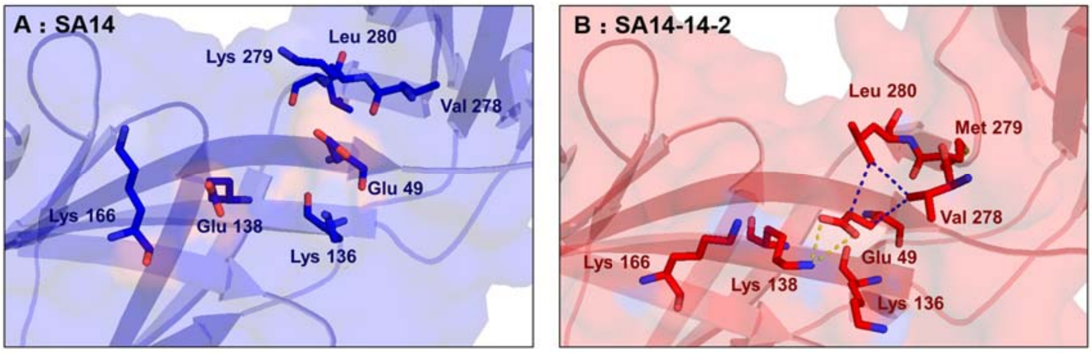

Figure S2. The ionic networks of amino acids at positions 138 and 279

(A) and (B), Surrounding around residue 138 of JEV SA14 and SA14-14-2 E protein ectodomain in the same orientation. Dot lines in blue shows the hydrophobic patch formed by Glu49, Val278 and Leu280. The dot lines in yellow show the ionic connection between residues Lys138 and Glu49. Residues of JEV SA14 are marked in dark blue and JEV SA14-14-2 in red.

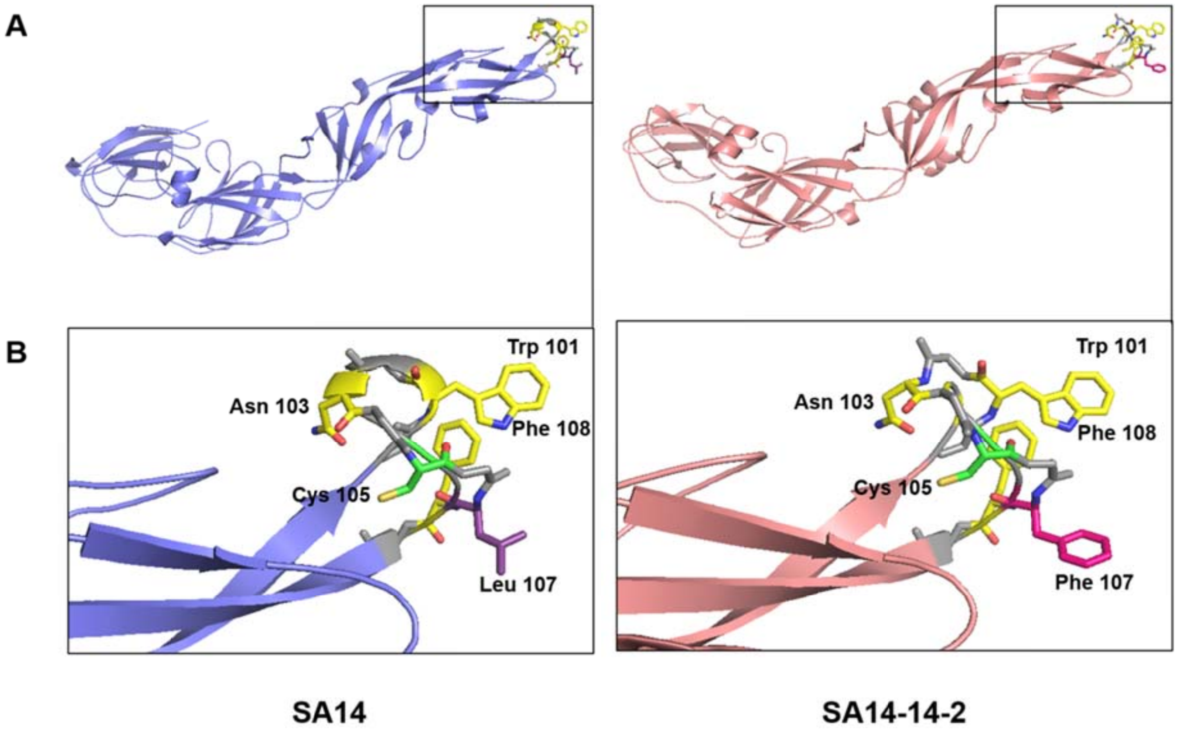

Figure S3. Fusion loops of JEV SA14 and SA14-14-2 E proteins

(A) The structures of SA14 (left) and SA14-14-2 (right) E proteins. The square marks are highlighting the key residues on the fusion loops.

(B) Magnification views of residues on the fusion loop of SA14 and SA14-14-2 E

proteins. They are Trp101, Asn103, Cys105, Phe108 and a mutated site Leu107Phe. Hydrophobic amino acids Asn103, Trp101 and Phe108 are shown in yellow sticks and Cys105 in green. Leu107 in SA14 is purple and Phe107 in SA14-14-2 is dark pink. Glycine is shown as grey.

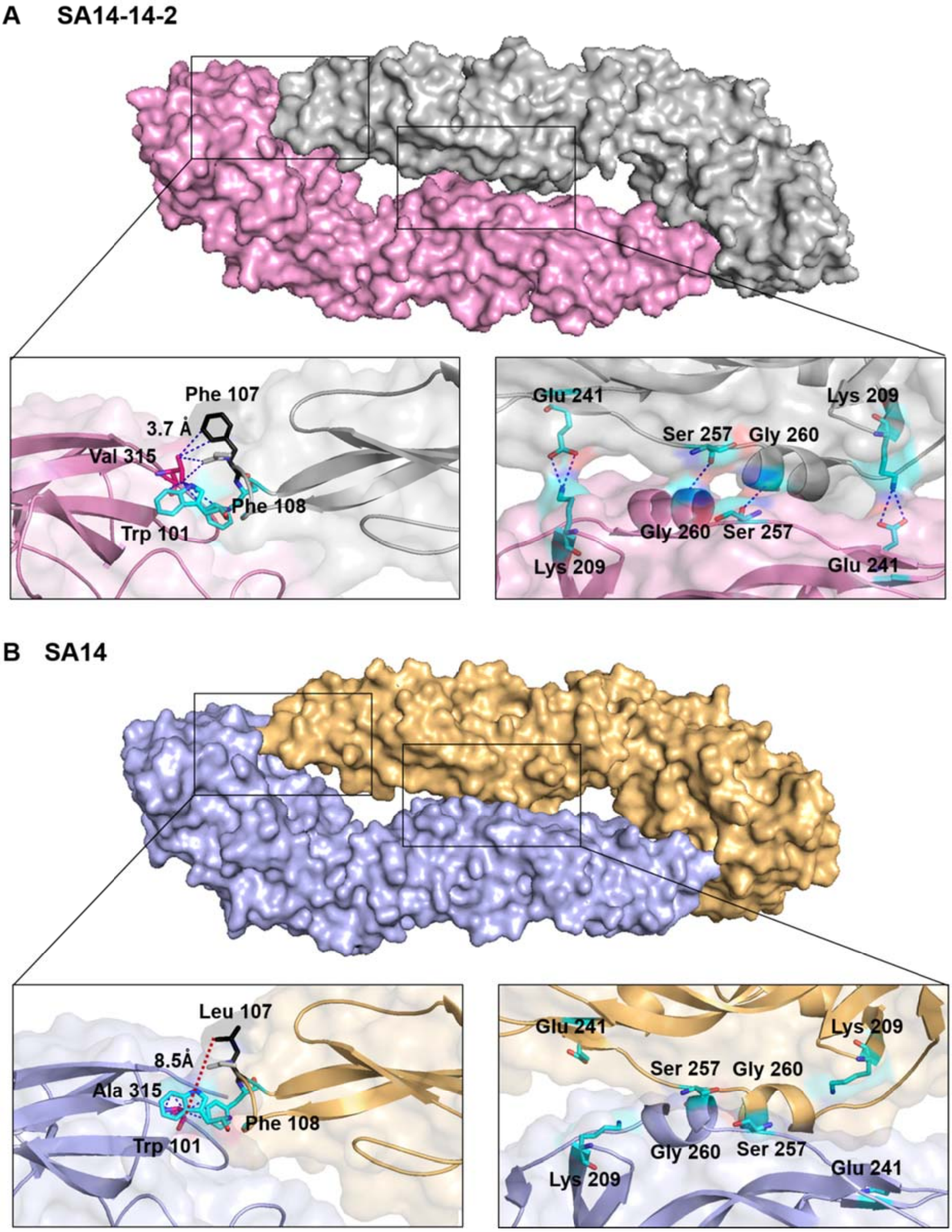

Figure S4. Dimer structural interfaces of JEV SA14 and SA14-14-2 E proteins

(A) The dimer interfaces of JEV SA14-14-2 E protein and the magnification views between two monomers.

(B) the dimer model of JEV SA14 is as the ensemble which calculated by PISA. The dimer interfaces of JEV SA14-14-2 E protein and the magnification views between two monomers. The amino acid 315 and 107 sites are displayed as dark pink and black. The fusion loop of one JEV SA14 E protein molecule interacts with the pocket of another, whereas the other side is farther away. The distance between Val315 and Phe107 in of JEV SA14-14-2 is 3.7 Å and that between Leu107 and Ala315 in JEV SA14 E protein is 8.5 Å.

1 Luca, V. C., AbiMansour, J., Nelson, C. A. & Fremont, D. H. Crystal structure of the Japanese encephalitis virus envelope protein. *Journal of virology* **86**, 2337-2346, doi:10.1128/jvi.06072-11 (2012).

2 Dai, L. *et al.* Structures of the Zika Virus Envelope Protein and Its Complex with a Flavivirus Broadly Protective Antibody. *Cell host & microbe* **19**, 696-704, doi:10.1016/j.chom.2016.04.013 (2016).

3 Kabsch, W. Automatic Processing of Rotation Diffraction Data from Crystals of Initially Unknown Symmetry and Cell Constants. *J Appl Crystallogr* **26**, 795-800 (1993).

4 McCoy, A. J. *et al.* Phaser crystallographic software. *Journal of applied crystallography* **40**, 658-674, doi:10.1107/s0021889807021206 (2007).

5 Murshudov, G. N. *et al.* REFMAC5 for the refinement of macromolecular crystal structures. *Acta crystallographica. Section D, Biological crystallography* **67**, 355-367, doi:10.1107/s0907444911001314 (2011).

6 Adams, P. D. *et al.* PHENIX: a comprehensive Python-based system for macromolecular structure solution. *Acta crystallographica. Section D, Biological crystallography* **66**, 213-221, doi:10.1107/s0907444909052925 (2010).

7 Emsley, P., Lohkamp, B., Scott, W. G. & Cowtan, K. Features and development of Coot. *Acta crystallographica. Section D, Biological crystallography* **66**, 486-501, doi:10.1107/s0907444910007493 (2010).

8 Yang, J. *et al.* Envelope Protein Mutations L107F and E138K Are Important for Neurovirulence Attenuation for Japanese Encephalitis Virus SA14-14-2 Strain. *Viruses* **9**,

doi:10.3390/v9010020 (2017).

9 Arroyo, J. *et al.* Molecular basis for attenuation of neurovirulence of a yellow fever Virus/Japanese encephalitis virus chimera vaccine (ChimeriVax-JE). *Journal of virology* **75**, 934-942, doi:10.1128/jvi.75.2.934-942.2001 (2001).

10 Gromowski, G. D., Firestone, C. Y. & Whitehead, S. S. Genetic Determinants of Japanese Encephalitis Virus Vaccine Strain SA14-14-2 That Govern Attenuation of Virulence in Mice. *Journal of virology* **89**, 6328-6337, doi:10.1128/jvi.00219-15 (2015).

11 Zhao, Z. *et al.* Characterization of the E-138 (Glu/Lys) mutation in Japanese encephalitis virus by using a stable, full-length, infectious cDNA clone. *The Journal of general virology* **86**, 2209-2220, doi:10.1099/vir.0.80638-0 (2005).

12 Sumiyoshi, H., Tignor, G. H. & Shope, R. E. Characterization of a highly attenuated Japanese encephalitis virus generated from molecularly cloned cDNA. *The Journal of infectious diseases* **171**, 1144-1151 (1995).

13 Nybakken, G. E. *et al.* Structural basis of West Nile virus neutralization by a therapeutic antibody. *Nature* **437**, 764-769, doi:10.1038/nature03956 (2005).

14 Huang, C. Y. *et al.* The dengue virus type 2 envelope protein fusion peptide is essential for membrane fusion. *Virology* **396**, 305-315, doi:10.1016/j.virol.2009.10.027 (2010).

15 Guirakhoo, F., Hunt, A. R., Lewis, J. G. & Roehrig, J. T. Selection and partial characterization of dengue 2 virus mutants that induce fusion at elevated pH. *Virology* **194**, 219-223, doi:10.1006/viro.1993.1252 (1993).

16 Wang, X. *et al.* Near-atomic structure of Japanese encephalitis virus reveals critical determinants of virulence and stability. **8**, 14, doi:10.1038/s41467-017-00024-6 (2017).

17 Aihara, S. *et al.* Identification of mutations that occurred on the genome of Japanese encephalitis virus during the attenuation process. *Virus genes* **5**, 95-109 (1991).
